# Donor selection for a second allogeneic stem cell transplantation in AML patients relapsing after a first transplant: a study of the Acute Leukemia Working Party of EBMT

**DOI:** 10.1038/s41408-019-0251-3

**Published:** 2019-11-18

**Authors:** Avichai Shimoni, Myriam Labopin, Jürgen Finke, Fabio Ciceri, Eric Deconinck, Nicolaus Kröger, Martin Gramatzki, Matthias Stelljes, Didier Blaise, Friedrich Stoelzel, Patrice Chevallier, Ernst Holler, Nathalie Fegueux, Mohamad Mohty, Arnon Nagler

**Affiliations:** 1 Division of Hematology and Bone Marrow Transplantation, Chaim Sheba Medical Center, Tel-Aviv University, Tel Aviv, Israel; 2 Acute Leukemia Working Party of EBMT, Paris, France; 3 Department of Medicine, Hematology-Oncology, University of Freiburg, Freiburg, Germany; 4 Hematology and Bone Marrow Transplantation Unit, IRCCS San Raffaele Scientific Institute, Milan, Italy; 5 Service d’Hématologie, Hopital Jean Minjoz, Besancon, France; 6 Department of Stem Cell Transplantation, University Medical Center Hamburg-Eppendorf, Hamburg, Germany; 7 Division of Stem Cell Transplantation & Immunotherapy, 2nd Medical Department, University Hospital Schleswig-Holstein, Campus Kiel, Kiel, Germany; 8 Department of Internal Medicine A, University of Muenster, Muenster, Germany; 9 Department of Hematology, Institut Paoli Calmettes, Marseille, France; 10 Department of Hematology/Oncology, Medical Clinic and Policlinic, University Hospital Carl Gustav Carus Dresden, Technical University Dresden, Dresden, Germany; 11 Clinical Hematology, Nantes University Hospital, Nantes, France; 12 Department of Hematology and Oncology, University Hospital Regensburg, Regensburg, Germany; 13 Department of Hematology, CHU Lapeyronie, Montpellier, France; 14 Department of Haematology, Saint Antoine Hospital, Paris, France

**Keywords:** Acute myeloid leukaemia, Medical research

## Abstract

Second allogeneic stem-cell transplantation (SCT2) is a therapeutic option for patients with AML relapsing after a first transplant. Prior studies have shown similar results after SCT2 from the same or different donor; however, there are limited data on second non-T-depleted haplo-identical transplant in this setting. We retrospectively analyzed SCT2 outcomes in 556 patients, median age 46 years, relapsing after first transplant given in CR1. Patients were divided into three groups based on SCT2 donor (donor2): same donor (*n* = 163, sib/sib-112, UD/UD-51), different matched donor (*n* = 305, sib/different sib-44, sib/UD-93, UD/different UD-168), or haplo-donor (*n* = 88, sib/haplo-45, UD/haplo-43). Two-year leukemia-free survival (LFS) rate after SCT2 was 23.5%, 23.7%, and 21.8%, respectively (*P* = 0.30). Multivariate analysis showed no effect of donor2 type on relapse: hazard ratio (HR) 0.89 (*P* = 0.57) and 1.11 (*P* = 0.68) for different donor and haplo-donor compared to same donor, respectively. However, donor2 did predict for non-relapse mortality (NRM) after SCT2: HR 1.21 (*P* = 0.50) and 2.08 (*P* = 0.03), respectively, and for LFS: HR 1.00 (*P* = 0.97) and 1.43 (*P* = 0.07), respectively. In conclusion, SCT2 with the same or different matched donor is associated with similar outcomes in patients with relapsed AML. Non-T-depleted haplo-identical transplant may be associated with higher NRM, similar relapse rate and with no better results in this setting.

## Introduction

Allogeneic hematopoietic stem-cell transplant (SCT) is a potentially curative treatment for acute myeloid leukemia (AML). Marked improvement in the rates of non-relapse mortality (NRM) after SCT has been achieved in the last decade^1^. However, relapse remains the main cause of treatment failure. There is no established standard of care therapy for patients relapsing after SCT. Patients can be treated with a spectrum of treatments including palliative care, withdrawal of immune-suppression therapy, low dose or intensive chemotherapy, hypomethylating agents or other targeted therapies, donor lymphocyte infusion (DLI), a second allogeneic transplant, or combinations of these therapies. However, the outcome of these patients remain poor, in particular in patients relapsing within the first 6 months after SCT^2^. In all, prolonged survival can be achieved only in patients achieving a second complete remission and supported by a form of cellular therapy such as DLI or second SCT^3^.

Several studies have shown that the major predictors of outcome after a second SCT are the duration of remission after the first SCT and the status of disease at the second SCT^4–13^. Advanced age and second SCT from an unrelated donor have also been described as predictors of inferior outcome. Most studies have not shown an advantage of switching to a different donor, attempting at enhancing a stronger or different graft-versus-leukemia (GVL) effect. However, there are so far limited data on the use of haplo-identical SCT as a second SCT in patients relapsing after a first SCT from a matched donor^14,15^.

The use of haplo-identical SCT has markedly increased in the last decade with the introduction of non-T-depleted platforms and in particular with the use of post-transplant cyclophosphamide^16^. Several studies have shown comparable outcomes of AML patients given first SCT from haplo-identical donors and unrelated and even matched sibling donors^17–19^. There is still controversy, whether haplo-identical SCT is associated with a stronger GVL effect than matched donor SCT^20,21^. If so, it is conceivable that haplo-identical SCT may result in better outcome in high-risk settings such as a second SCT after failure of a first SCT from an HLA-matched donor.

In the current study we show that a second SCT from a haplo-identical donor is not associated with improved outcome. It is not associated with stronger GVL, but NRM is increased in comparison with matched donor SCT, and therefore leukemia-free survival (LFS) is lower.

## Patients and methods

### Study design and data collection

This is a retrospective multicenter analysis. Data were provided and approved for this study by the acute leukemia working party (ALWP) of the European Society for Blood and Marrow Transplantation (EBMT). The study protocol was approved by the institutional review board at each site and complied with country-specific regulatory requirements. All patients provided written informed consent authorizing the use of their personal information for research purposes. Eligibility criteria included patients with de-novo or secondary AML who relapsed after allogeneic SCT from an HLA-matched sibling or unrelated donor, and were given a second SCT from the same or a different donor, in any disease status, between the years 2006 and 2016. Patients were required to engraft after SCT1, to relapse before SCT2 and to be given SCT2 within 300 days after relapse. Patients were divided into three groups based on the donor selected for SCT2: same donor group, different HLA-matched donor group, and second haplo-donor group. Haplo-identical donors were defined as two or more mismatches from a related donor. There was no haplo-identical unrelated donor. The EBMT database does not include enough information on kinship of haplo-identical donors. Most unrelated donors were 9–10 HLA matched. Variables collected included recipient and donor characteristics for both transplants, disease features, transplant related factors including drugs and total doses used in the conditioning regimen, and outcome variables.

### Conditioning regimens

The conditioning regimen was selected according to the participating center discretion. Dose intensity was defined according to standard criteria based on the reversibility and expected duration of cytopenia after SCT^22^. GVHD prophylaxis was selected according to the participating center policy and consisted of a calcineurin inhibitor (cyclosporine A or tacrolimus) with short-term methotrexate or mycophenolate mofetil in most HLA-matched transplants. Antithymocye globulin (ATG) was allowed according to the participating center policy. Haplo-identical transplants were all non-T cell depleted based on either post-transplant cyclophosphamide (PTCy) or ATG. No ex vivo manipulation was allowed for any SCT.

### Evaluation of outcomes

Disease relapse was defined according to standard hematological criteria. NRM was defined as death of any cause in the absence of prior disease recurrence. LFS was defined as survival without relapse. Overall survival (OS) was calculated from the day of SCT until death of any cause or last follow-up. The events for analysis of GVHD-free relapse-free survival (GRFS) were relapse, death of any cause, acute GVHD grade III–IV, or extensive chronic GVHD, which ever occurred first. Patients with no event were censored at last contact. Acute and chronic GVHD were graded according to standard criteria.

### Statistical analysis

The primary end-point of the study was LFS after SCT2. Secondary endpoints included acute and chronic GVHD, NRM, relapse incidence, OS, and GRFS. All outcomes were measured from the time of stem cell infusion. The three donor groups were compared by the Chi-square method for qualitative variables, and Mann–Whitney test for continuous parameters. LFS, OS, and GRFS were estimated using the Kaplan–Meier method^23^ while NRM, relapse, and GVHD were estimated using cumulative incidence analysis considering competing risks^24^. Univariate comparisons were done using the log-rank test for LFS and OS, and Gray’s test for GVHD, relapse incidence, and NRM. For all univariate analyses, continuous variables were categorized and the median used as a cut-off point. Multivariate analyses were performed using Cox proportional hazards. Variables were included in the multivariate model if they were conceptually important or if they differ in term of distribution between the three groups. Results are expressed as hazard ratio (HR) with 95% confidence interval. To test for a center effect, we introduced a random effect or frailty for each center into the model. All *P* values were two-sided and values <0.05 were considered statistically significant. Statistical analyses were performed with SPSS 24.0 (Inc., Chicago) and R 3.4.1 software packages (R Core Team (2017). R: A language and environment for statistical computing. R Foundation for Statistical Computing, Vienna, Austria. URL https://www.R-project.org/).

## Results

### Patient characteristics

The study included 556 patients with AML relapsing after a first allogeneic SCT (SCT1) given in CR1 from an HLA-matched sibling (sib, *n* = 294) or a matched unrelated donor (UD, *n* = 262) and given SCT2. Patient characteristics are outlined in Table 1. The median age at SCT2 was 46 years (range, 20–73). Two hundred and forty-six patients were in CR2 (44%) and 309 had active leukemia (55%) at the time of SCT2. The conditioning regimen in SCT1 was myeloablative (MAC, 66%) or reduced intensity (RIC, 34%), and was 41% and 59%, respectively, in SCT2. The combination of regimen intensities in both transplants (SCT1/SCT2, respectively) were MAC/MAC (29%), MAC/RIC (37%), RIC/MAC (11%), and RIC/RIC (23%). The specific conditioning regimens are given in Table 2. The most commonly used MAC regimens for SCT1 were BuCy (44%) and TBI- based (28%). The MAC regimens were more heterogeneous in SCT2 and were mostly TBI-based (22%) or treosulfan-based (15%). Only one patient had the same MAC regimen in both transplants. The most commonly used RIC regimens for SCT1 was FluBu (37%) or FluMel (22%). Similarly, the RIC regimens were more heterogeneous in SCT2 and were mostly low-dose TBI-based (18%) or Flu/Mel (17%). Twenty patients (17%) who received RIC for both SCT1 and SCT2 had the same RIC regimen in both transplants, mostly low-dose TBI-based. Nineteen percent of all patients had acute GVHD grade II–IV and 26% had chronic GVHD after SCT1 and before relapse. Patients were divided into three groups based on the donor selected for SCT2. The combinations of donors for the SCT1 and SCT2 were: (1) same donor group (*n* = 163, sib/sib-112, UD/UD-51), (2) different HLA-matched donor (*n* = 305, sib/different sib-44, sib/UD-93, UD- different UD-163), and (3) second haplo-donor (*n* = 88, sib/relate haplo-45, UD/related haplo-43). All haploSCT were non-T-depleted. There were some differences between the three groups in the timing of relapse and SCT2. The median time from SCT1 to relapse was similar: 10.6, 12.5, and 9.3 months, respectively (*P* = 0.14), and 36%, 28%, and 35%, respectively, relapsed within 6 months of SCT1 (*P* = 0.13). However, the median time from relapse to SCT2 was shorter for the same donor group: 2.8, 3.7, and 3.5 months, respectively (*P* < 0.001), and the median time between SCT1 and SCT2 was longer for the different donor group: 14.3, 17.5 and 13.8 months, respectively (*P* = 0.03). There was no difference between the groups in patient age, gender, and disease status at SCT2. The Karnofsky performance status at SCT2 was better for the haplo-identical donor group (*P* = 0.001). The conditioning regimen intensity for SCT1 or SCT2 was similar. However, more patients in the different matched group were given in vivo T cell depletion (mostly ATG) in SCT2. All haplo-identical SCT2 were non-T depleted; 70% were given PTCy, 25% ATG, and 5% both. The median follow-up was 52.0 months (0.9–131.8), 30.5 months (1.0–135.1), and 33.0 (1.0–73.9) following the three donor groups, respectively (*P* = 0.05).

**Table 1:** Patient characteristics.

	Donor for the second SCT			
	Same donor (*n* = 163)	Different matched donor (*n* = 305)	Haplo-identical (*n* = 88)	*P* value
Age at SCT2 (years, range)	46 (20–73)	48 (20–69)	45 (20–71)	0.40
Gender (male)	52%	51%	41%	0.67
Secondary AML	16%	16%	15%	0.97
Cytogenetics				
Good	5%	3%	1%	0.009
Intermediate	46%	39%	26%	
Poor	18%	15%	23%	
Missing	31%	43%	50%	
Conditioning SCT1				
MAC	65%	66%	64%	0.90
RIC	35%	34%	36%	
Donor SCT1				
Sib	69%	45%	51%	<0.001
UD	31%	55%	49%	
10/10	81%	78%	62%	
≤9/10	19%	22%	38%	
aGVHD after SCT1	22.8%	15.7%	20.9%	0.15
cGVHD after SCT1	26.2%	26.8%	23.9%	0.89
Time SCT1 → relapse (months, range)	10.6 (1-129.9)	12.5 (0.6-236.2)	9.3 (1.1-80.8)	0.14
Time <6 months	36%	28%	35%	0.13
Time relapse → SCT2	2.8 (0.5-11.5)	3.7 (0.5-11.5)	3.5 (0.7–9.7)	<0.001
Time SCT1 → SCT2	14.3 (1.7-134.8)	17.5 (1.9-244.5)	13.8 (3.1-88.2)	0.03
Donor SCT1/ SCT2	Sib/Sib (*n* = 112) UD/UD (*n* = 51)	Sib/d-Sib (*n* = 44) Sib/UD (*n* = 93) UD/d-UD (*n* = 168) New UD 10/10 77% ≤ 9/10 23%	Sib/haplo (*n* = 45) UD/haplo (*n* = 43)	
Donor gender (male)	66%	64%	64%	0.84
F → M	19%	15%	14%	0.52
Patient CMV (pos)	66%	61%	72%	0.18
Donor CMV (pos)	58%	45%	59%	0.02
Disease status SCT2				
CR2	45%	45%	44%	0.96
Active leukemia	56%	55%	56%	
KPS at SCT2 < 90	53%	44%	27%	0.001
Conditioning SCT2				
MAC	45%	39%	40%	0.48
RIC	55%	61%	60%	
in vivo TCD	24%	68%	30%	<0.001
Year of SCT2	2011 (2006–2016)	2013 (2006–2016)	2014 (2006–2016)	<0.001

*SCT* stem cell transplantation, *SCT1* first SCT, *SCT2* second SCT, *MAC* myeloablative conditioning, *RIC* reduced intensity conditioning, *aGVHD* acute GVHD grade II–IV, *cGVHD* chronic GVHD (all grades), *Sib* sibling donor, *d-sib* different sibling donor, *UD* unrelated donor, *d-UD* different unrelated donor, *haplo* haplo-identical donor, *F* *→* *M* female donor to male recipient, *pos* positive, *KPS* Karnofsky performance score, *in vivo TCD* T cell depletion (mostly ATG, does not include post-transplant cyclophosphamide)

**Table 2:** Conditioning regimens used in SCT1 and SCT2.

	Myeloablative regimens		Reduced- intensity regimens	
	MAC 1	MAC 2	RIC 1	RIC 2
BuCy	44%	13%		
FluBu^a^	17%	9%	37%	14%
Melphalan-based	3%	4%	22%	17%
Thiotepa-based^b^	0.3%	12%	3%	14%
Treosulfan-based	5%	15%	11%	8%
TBI-based^c^	28%	22%	15%	18%
FLAMSA (sequential)^d^	2%	7%	9%	10%
Others	1%	17%	4%	19%

Abbreviations as in Table 1. *MAC 1* myeloablative conditioning in second transplant, *MAC 2* myeloablative conditioning in second transplant, *RIC 1* reduced intensity conditioning in first transplant, *RIC 2* reduced intensity conditioning in second transplant, *BuCy* high-dose busulfan and cyclophosphamide, *TBI* total body radiation. ^a^Fludarabine and busulfan. Mostly 4 days of busulfan (total 12.8 mg/kg of IV formulation) for MAC and 2 days (6.4 mg/kg) for RIC ^b^Mostly the TBF regimen consisting of fludarabine, thiotepa, and busulfan in myeloablative or reduced-intensity doses as previously reported ^c^Mostly 12 cGy for MAC and 2–4 cGy for RIC with other agents ^d^Sequential therapy of FLAMSA (or other induction regimens) with MAC or RIC subsequent transplant conditioning

### Acute and chronic GVHD

The cumulative incidence of acute GVHD grade II–IV was 35.9% (95% CI, 28.0–43.9), 32.7% (95% CI, 27.2–38.3), and 20.1% (95% CI, 12.1–29.6) in the same donor, different matched donor, and haplo-identical donor groups, respectively (*P* = 0.05). Multivariate analysis identified acute GVHD after SCT1 as a risk factor for acute GVHD after SCT2 [hazard ratio (HR), 2.36 (95% CI, 1.46–3.82, *P* < 0.001]. Advanced age [HR, 0.84 (95% CI, 0.70–1.00), *P* = 0.05] and the use of in vivo T cell depletion [HR, 0.44 (95% CI, 0.28–0.71), *P* < 0.001] were associated with a lower risk for acute GVHD after SCT2 (data not shown). The second donor type group was not predictive.

The cumulative incidence of chronic GVHD was 36.6% (95% CI, 28.1–45.0), 31.0% (95% CI, 25.1–37.1), and 25.1% (95% CI, 15.0–36.5) in the same donor, different matched donor, and haplo-identical donor groups, respectively (*P* = 0.42). Multivariate analysis identified chronic GVHD after SCT1 as a risk factor for chronic GVHD after SCT2 [HR, 2.21 (95% CI, 1.40–3.451), *P* < 0.001]. The use of in vivo T cell depletion [HR, 0.66 (95% CI, 0.43–1.04), *P* = 0.07] was associated with a lower risk chronic GVHD after SCT2 (data not shown). The second donor type group was not predictive.

### Non-relapse mortality

**Fig. 1:**
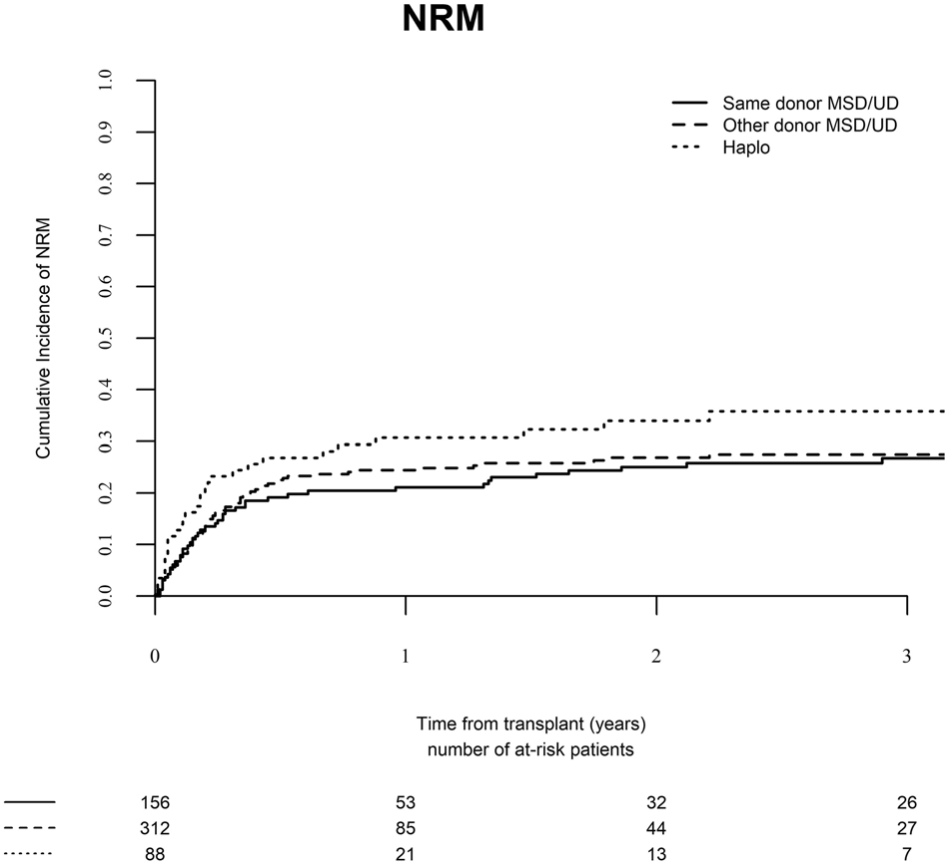
Non-relapse mortality (NRM) after second stem cell transplantation from the same donor, a different matched donor, or a haplo-identical donor.

The 2-year NRM rate was 25.1% (95% CI, 18.6–32.0), 26.9% (95% CI, 21.8–32.3), and 33.9% (95% CI, 23.7–44.4) in the same donor, different matched donor, and haplo-identical donor groups, respectively (Fig. 1, *P* = 0.28). Among the various causes of NRM there was a trend for higher cumulative incidence of death from infection in the haplo-identical group, rates been 10.6% (95% CI, 6.3–16.2), 17.1% (95% CI 12.9–21.8), and 20.7% (95% CI 12.6–30.2), respectively (*P* = 0.08). The cumulative incidence of death due to GVHD was not different between the groups, 7.4% (95% CI, 3.9–12.4), 10.9% (95% CI 7.4–15.1), and 9.3% (95% CI 4.0–17.4), respectively (*P* = 0.59). Table 3 outlines the multivariate analysis of predicting factors for NRM after SCT2. Advanced age [HR 1.39 (95% CI, 1.16–1.67), *P* = 0.0003] and SCT2 from haplo-identical donors [HR 2.08 (95% CI, 1.09–3.99), *P* = 0.03] were associated with increased risk, while MAC during SCT1 was associated with a lower risk of NRM after SCT2 [HR 0.63 (95%CI, 0.40–1.01), *P* = 0.06].

**Table 3:** Multivariate analysis of factors predicting relapse and non-relapse mortality.

	Relapse		NRM	
	HR	*P* value	HR	*P* value
Age (per 10 years)	0.96 (0.85–1.09)	0.53	1.39 (1.16–1.67)	0.0003
Donor SCT1 UD	0.73 (0.53–1.00)	0.05	1.34 (0.86–2.08)	0.20
Conditioning SCT1 MAC	1.21 (0.86–1.70)	0.28	0.63 (0.40–1.02)	0.06
aGVHD after SCT1	0.91 (0.61–1.37)	0.66	1.28 (0.78–2.08)	0.33
cGVHD after SCT1	0.69 (0.47–0.99)	0.05	1.43 (0.90–2.28)	0.13
SCT1 → relapse (<6 months)	1.47 (1.04–2.08)	0.03	1.49 (0.91–2.45)	0.12
Relapse → SCT2 (>median)	1.03 (0.97–1.09)	0.34	0.97 (0.89–1.06)	0.53
Donor SCT2				
Same donor	1.00		1.00	0.50
Different matched	0.89 (0.61–1.31)	0.57	1.21 (0.69–2.13)	0.03
Haplo-identical	1.11 (0.68–1.81)	0.68	2.08 (1.09–3.99)	
F → M	0.92 (0.61–1.40)	0.71	1.16 (0.66–2.04)	0.61
Disease status SCT2 CR	0.62 (0.46–0.84)	0.002	0.80 (0.53–1.21)	0.29
Conditioning SCT2 MAC	0.75 (0.55–1.03)	0.07	1.25 (0.83–1.87)	0.30
Patient CMV+	1.02 (0.75–1.40)	0.90	1.01 (0.65–1.57)	0.96
Donor CMV+	0.94 (0.69–1.29)	0.70	1.06 (0.69–1.62)	0.79
in vivo TCD	1.35 (0.97–1.87)	0.07	1.13 (0.72–1.77)	0.60
Year of SCT2	0.98 (0.93–1.03)	0.39	0.97 (0.91–1.04)	0.44
Center effect		0.32		0.92

Abbreviations as in Tables 1 and 2. *NRM* non-relapse mortality, *HR* hazard ratio

### Relapse

**Fig. 2:**
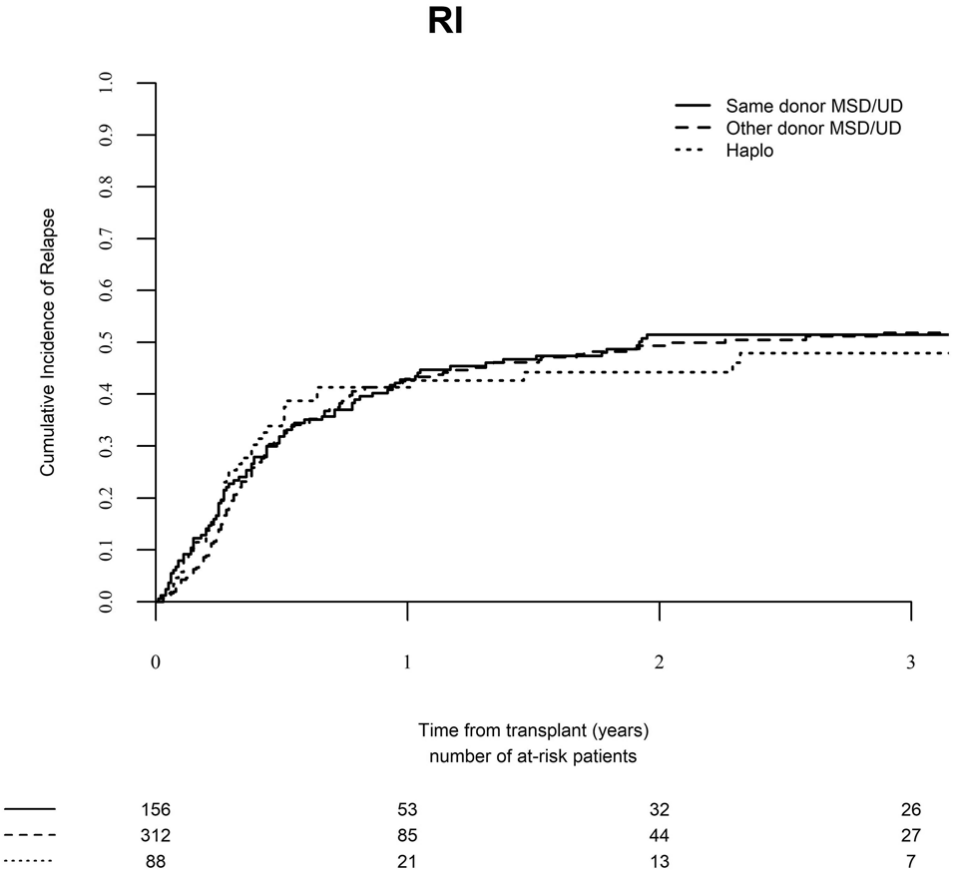
Relapse incidence (RI) after second stem cell transplantation from the same donor, a different matched donor, or a haplo-identical donor.

The 2-year relapse rate was 51.5% (95% CI, 43.3–59.0), 49.3% (95% CI, 43.1–55.3), and 44.2% (95% CI, 33.2–54.7) in the same donor, different matched donor, and haplo-identical donor groups, respectively (Fig. 2, *P* = 0.90). Multivariate analysis identified relapse within 6 months of SCT1 as associated with higher risk of relapse after SCT2 [HR 1.47 (95% CI, 1.04–2.08), *P* = 0.03]. Unrelated donor at SCT1 [HR 0.73 (95% CI, 0.53–1.00), *P* = 0.05], chronic GVHD after SCT1 [HR 0.69 (95% CI, 0.47–0.99), *P* = 0.05], and SCT2 in CR2 [HR 0.62 (95% CI, 0.46–0.84), *P* = 0.002] were factors associated with reduced risk of relapse after SCT2 (Table 3). The second donor type group was not predictive.

### Leukemia-free and overall survival

**Fig. 3:**
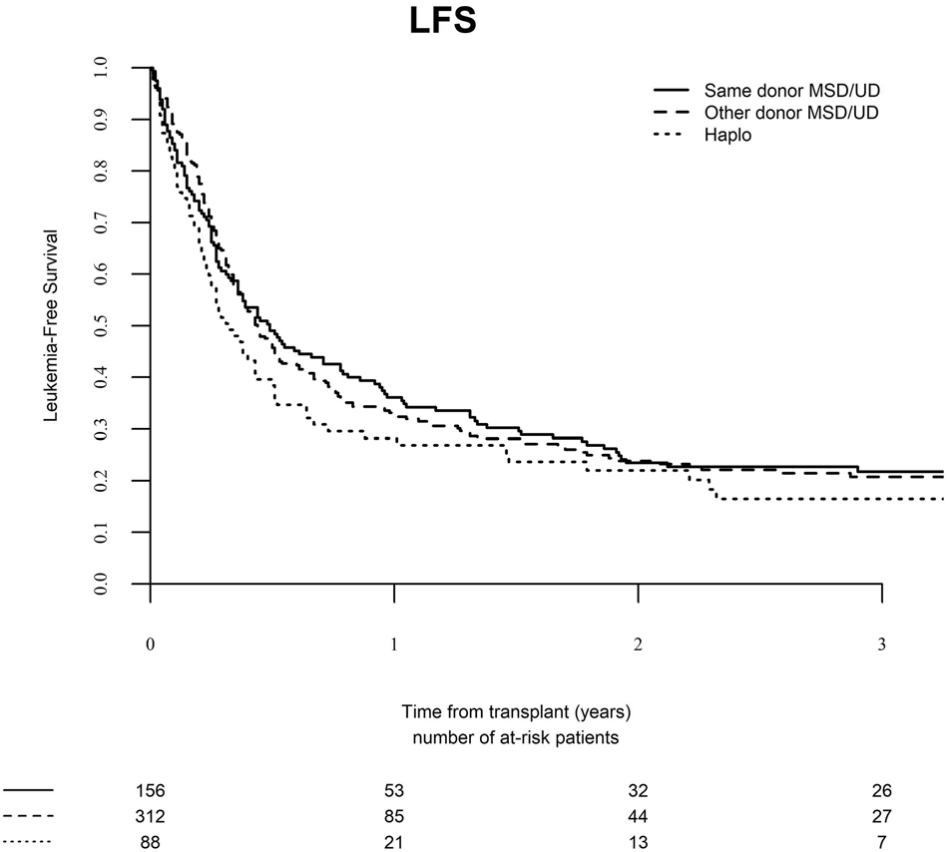
Leukemia-free survival (LFS) after second stem cell transplantation from the same donor, a different matched donor, or a haplo-identical donor.

**Fig. 4:**
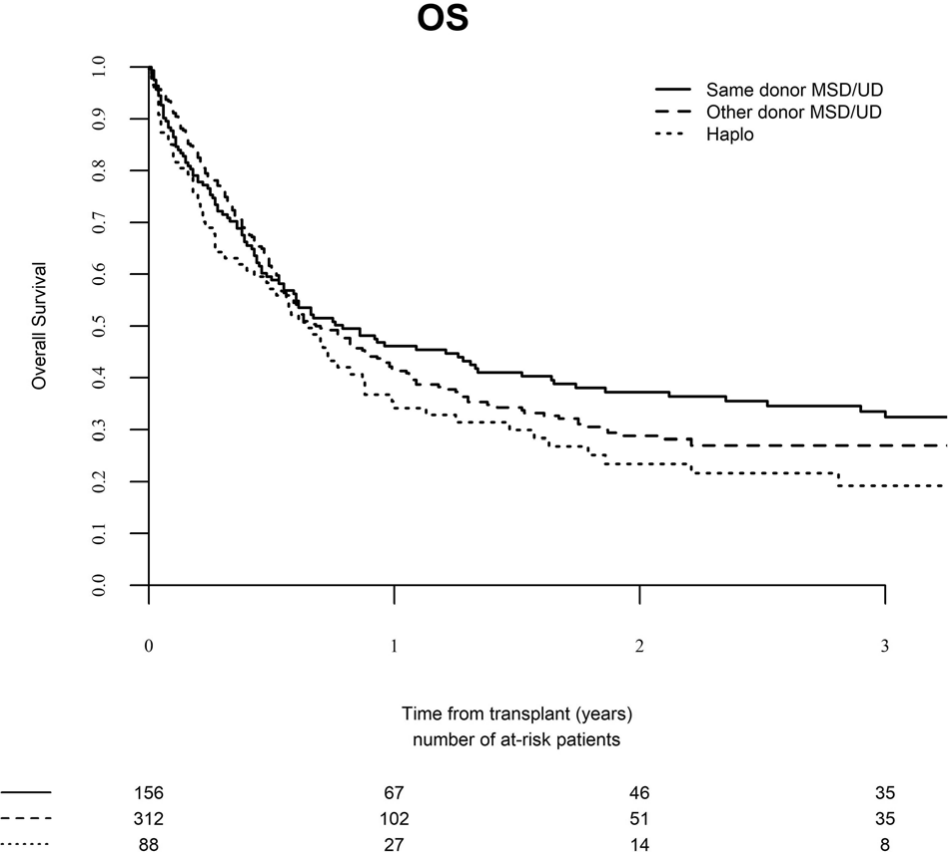
Overall Survival (OS) after second stem cell transplantation from the same donor, a different matched donor, or a haplo-identical donor.

The median follow-up after SCT2 was 52 months (range, 0.9–131.8), 30.5 months (range, 1–135.1), and 33.0 months (range, 1–73.9) following the three donor type groups, respectively (*P* = 0.05). One hundred and eighty-seven patients are alive and 369 have died. The major causes of death were disease recurrence (*n* = 181, 49%), GVHD (*n* = 48, 13%), infection (*n* = 87, 24%), veno-occlusive disease of the liver (*n* = 10, 3%), and others (*n* = 43, *n* = 12%). The 2-year LFS rate was 23.5% (95% CI, 16.8–30.2), 23.7% (95% CI, 18.3–29.1), and 21.8% (95% CI, 12.6–31.1) in the three groups, respectively (Fig. 3, *P* = 0.30). The 2-year OS rate was 36.4% (95% CI, 28.6–44.3), 28.7% (95% CI, 22.8–4.5), and 23.3% (95% CI, 13.7–33.0) in the three groups, respectively (Fig. 4, *P* = 0.21).

Table 4 outlines the multivariate analysis of predicting factors for LFS after SCT2. Relapse within 6 months of SCT1 [HR 1.46 (95% CI, 1.09–1.95), *P* = 0.01], advanced age [HR 1.10 (95% CI, 0.99–1.22), *P* = 0.07], and SCT2 from haplo-identical donors [HR 1.43 (95% CI, 0.96–2.13), *P* = 0.07] were associated with inferior LFS, while SCT2 in CR2 was associated with better LFS after SCT2 [HR 0.67 (95% CI, 0.52–0.87), *P* = 0.002]. Similar factors predicted for 2-year OS with HR 1.50 (95% CI, 1.11–2.03), *P* = 0.008), 1.13 (95% CI, 1.02–1.26), *P* = 0.03), 1.61 (95% CI, 1.06–2.544), *P* = 0.03), and 0.62 (95% CI, 0.48–0.81), *P* = 0.0004), respectively.

**Table 4:** Multivariate analysis of factors predicting LFS and OS.

	LFS		OS	
	HR	*P* value	HR	*P* value
Age (per 10 years)	1.10 (1.00–1.22)	0.07	1.13 (1.02–1.26)	0.03
Donor SCT1 UD	0.90 (0.70–1.17)	0.43	0.95 (0.72–1.24)	0.69
Conditioning SCT1 MAC	0.95 (0.72–1.26)	0.71	0.95 (0.71–1.27)	0.72
aGVHD after SCT1	1.06 (0.77–1.45)	0.72	1.14 (0.82–1.58)	0.43
cGVHD after SCT1	0.89 (0.67–1.20)	0.46	0.93 (0.68–1.26)	0.64
SCT1 → relapse (<6 months)	1.46 (1.09–1.95)	0.01	1.50 (1.11–2.03)	0.008
Relapse → SCT2 (>median)	1.01 (0.96–1.06)	0.66	1.00 (0.95–1.05)	0.87
Donor SCT2				
Same donor	1.00	0.97	1.00	0.31
Different matched	1.00 (0.72–1.37)	0.07	1.20 (0.85–1.70)	0.03
Haplo-identical	1.43 (0.96–2.13)		1.61 (1.06–2.44)	
F → M	1.01 (0.72–1.41)	0.96	1.08 (0.76-1.54)	0.68
Disease status SCT2 CR	0.67 (0.52–0.87)	0.002	0.62 (0.48–0.81)	0.0004
Conditioning SCT2 MAC	0.93 (0.73–1.20)	0.59	0.97 (0.75–1.25)	0.81
Patient CMV+	1.02 (0.79–1.32)	0.88	1.01 (0.77–1.32)	0.96
Donor CMV+	1.00 (0.78–1.29)	0.99	1.06 (0.81–1.38)	0.68
In vivo TCD	1.26 (0.96–1.64)	0.10	1.31 (0.99–1.73)	0.06
Year of SCT2	0.98 (0.94–1.02)	0.28	0.97 (0.93–1.01)	0.14
Center effect		0.30		0.32

Abbreviations as in Tables 1–3. *LFS* leukemia-free survival, *OS* overall survival

The 2-year composite outcome of GRFS was 14.1% (95% CI, 8.5–19.6), 17.0% (95% CI, 12.2–21.7), and 13.7% (95% CI, 6.0–21.5) in the same donor, different matched donor, and haplo-identical donor groups, respectively (*P* = 0.16). Multivariate analysis identified SCT2 in CR2 [HR 0.66 (95% CI, 0.53–0.84), *P* = 0.0006] as associated with better GRFS after SCT2 (data not shown). Haplo-identical donor was associated with a trend for inferior GRFS [HR 1.43 (95% CI, 0.98–2.10), *P* = 0.07].

## Discussion

A second allogeneic SCT is a valid treatment in relapsed AML after a first SCT. In the current study including a relatively large cohort of patients with AML, approximately 25% remained leukemia-free 2 years after SCT2, about 25% died of NRM causes and about 50% relapsed again after SCT2. We did not find a difference in SCT2 outcome between same or different matched donors (related or unrelated). However, second haplo-identical donors were associated with higher NRM and lower LFS.

Historically, second transplants were performed mostly from siblings, using the same donor and mostly myeloablative conditioning. Eapen et al. reported the first large series of 279 patients with acute and chronic leukemia having a second SCT from a sibling donor (in 15% a different donor). The 5-year rates of relapse, NRM, and LFS were 42%, 30%, and 28%, respectively^4^. Younger age, relapse beyond 6 months of the first SCT and SCT2 in CR were the most important prognostic factors for survival, but a change of donor did not result in better outcome. With the improvement in HLA typing and donor registries and with the introduction of RIC regimens the use of second SCT from unrelated donors and after a first SCT from unrelated donors markedly increased^5,7,12,13^. Christopeit et al.^7^ reported the first large study including unrelated donors for a second SCT (104 of 179 second transplants)^7^. The 2-year OS after SCT2 was 25%, 39% after sibling, and 19% after unrelated donor SCT2). Similarly, the favorable prognostic factors were longer prior remission, CR at SCT2 and SCT1 from a sibling. Selecting a new donor did not change outcome in the entire group. There was a better outcome when changing from a sibling to a different sibling but this was based on a very small group. There was also an advantage in changing MUD to a different MUD that was limited to patients with no prior chronic GVHD. Ruuto et al. summarized 2632 second transplants for relapse of various hematological malignancies reported to the EBMT^9^. The 5-year OS was 20%. There was a trend for lower risk of relapse when changing a donor; however, this was counter balanced by a trend for higher NRM, and overall survival was similar. Most other studies have also failed to show a difference in outcome with donor change^5,6,8,11,12^.

Switching to a haplo-identical donor after a matched donor may theoretically offer an advantage^14,15^. HLA disparity can contribute to allo-reactivity and GVL. The frequency of donor T cell precursors directed against minor histocompatibility antigens or leukemia-specific antigens that mediate GVL in the matched donor transplant setting is several logs lower than the frequency of T cells directed against major HLA differences^25^. Loss of the unshared haplotype is a common mechanism for leukemia immune escape after haplo-identical donor transplant^26^. Therefore, switching to a different haplo-identical donor may be an effective strategy in a second transplant^21^. Using a haplotype mismatched donor may also offer new targets for GVL after failure of a first matched transplant and improve outcome^21^. However, the current study fails to show better GVL with a second haplo-donor, and since a second haplo-identical transplant was associated with more NRM, outcome was inferior. Mismatched HLA can be a target for natural killer (NK) cell allo-reactivity in T cell-depleted haplo-identical transplant with no post-transplant immune-suppression^27^ but this NK cell activity is controversial in the T cell-replete setting^28^. The use of post-transplant cyclophosphamide may even abrogate T cell precursors that are rapidly dividing after the mismatched transplant such as those against major HLA antigens, more than those that are less frequent. However, a more precise and individualized study of HLA antigens expression by leukemia blasts at relapse could guide in the future a personalized choice of donor for SCT2 searching for specific HLA-mismatches towards a disease immune-edited by previous mismatched donor.

A haplo-identical donor is more rapidly available than when searching for a different unrelated donor. Despite that, in the current study the time from relapse to SCT2 was similar between different matched donor (mostly unrelated) and haplo-identical donors, suggesting that a haplo-identical donor was chosen only after failure in allocating a different matched donor. This may explain in part the higher NRM with haplo-identical donors. Randomized prospective studies comparing these different donors are needed to overcome these limitations of retrospective registry analysis.

Similarly to previous studies, the major factors predicting outcome after SCT2 were the biological aggressiveness of the underlying leukemia as reflected by the time to relapse after SCT1 and the ability to achieve a second remission prior to SCT2 (refs. ^4–15^). The conditioning regimen used in the first or second transplant had no impact on subsequent survival.

Chronic GVHD after SCT1 was associated with a lower rate of relapse after SCT2 although not with statistically significant improvement in OS. Two recent studies reported similar observations. The pediatric diseases working party of the EBMT determined in a group of 373 children with acute leukemia following a second SCT that the prognostic factors for improved survival were longer duration (>1 years) between transplantations, chronic GVHD after the first SCT, CR at SCT2 (in ALL), and age >12 years (in AML)^8^. Prior chronic GVHD was independently associated with reduced rates of relapse and NRM and improved survival. The ALWP of EBMT reported a retrospective comparison of second SCT and DLI in the treatment of post-transplant relapse in AML. Prior chronic GVHD improved LFS while not increasing NRM during further therapy^11^. Several studies have shown a beneficial effect of chronic GVHD in reducing relapse after SCT^29^ including SCT2 (refs. ^10,12^). It seems this effect carries over after relapse during further treatment with SCT2 or DLI. The conditioning regimen and immune-suppressive therapy after SCT2 did not eliminate this GVL effect, or alternatively a supportive cytokine or allo-immune milieu induced by prior chronic GVHD continues to influence donor cells whether from the same or a different donor. However, not all studies reported a similar effect of chronic GVHD. Ruuto et al. reported, in a very large series of second SCT for various hematological malignancies, that prior chronic GVHD was associated with higher NRM and lower survival after SCT2 (ref. ^9^). More studies are needed to confirm if this observation is limited to patients with acute leukemia or what is the validity of this finding. There are no data supporting donor selection based on prior GVHD. Patients with prior GVHD had similar outcomes with same or different donors^9^. In the current study we were unable to perform a subset analysis based on prior GVHD due to patient numbers and there was no interaction between second donor and prior GVHD.

In conclusion, a second SCT from the same or a different matched donor is a valid therapeutic option for patients with AML relapsing after a first matched donor transplant with equivalent outcome. A second transplant from a haplo-identical donor was not associated with better outcome than other donor sources in this setting.
